# Predicting malignant risk of ground-glass nodules using convolutional neural networks based on dual-time-point ^18^F-FDG PET/CT

**DOI:** 10.1186/s40644-025-00834-8

**Published:** 2025-02-18

**Authors:** Yuhang Liu, Jian Wang, Bulin Du, Yaming Li, Xuena Li

**Affiliations:** Department of Nuclear Medicine, The First Hospital of China Medical University, No. 155 Nanjing St, Shenyang, 110001 China

**Keywords:** Convolutional neural network, Lung, Ground-Glass nodules, Malignant risk, Dual-time-point ^**18**^**F-FDG PET/CT**

## Abstract

**Background:**

Accurately predicting the malignant risk of ground-glass nodules (GGOs) is crucial for precise treatment planning. This study aims to utilize convolutional neural networks based on dual-time-point ^18^F-FDG PET/CT to predict the malignant risk of GGOs.

**Methods:**

Retrospectively analyzing 311 patients with 397 GGOs, this study identified 118 low-risk GGOs and 279 high-risk GGOs through pathology and follow-up according to the new WHO classification. The dataset was randomly divided into a training set comprising 239 patients (318 lesions) and a testing set comprising 72 patients (79 lesions), we employed a self-configuring 3D nnU-net convolutional neural network with majority voting method to segment GGOs and predict malignant risk of GGOs. Three independent segmentation prediction models were developed based on thin-section lung CT, early-phase ^18^F-FDG PET/CT, and dual-time-point ^18^F-FDG PET/CT, respectively. Simultaneously, the results of the dual-time-point ^18^F-FDG PET/CT model on the testing set were compared with the diagnostic of nuclear medicine physicians.

**Results:**

The dual-time-point ^18^F-FDG PET/CT model achieving a Dice coefficient of 0.84 ± 0.02 for GGOs segmentation and demonstrating high accuracy (84.81%), specificity (84.62%), sensitivity (84.91%), and AUC (0.85) in predicting malignant risk. The accuracy of the thin-section CT model is 73.42%, and the accuracy of the early-phase ^18^F-FDG PET/CT model is 78.48%, both of which are lower than the accuracy of the dual-time-point ^18^F-FDG PET/CT model. The diagnostic accuracy for resident, junior and expert physicians were 67.09%, 74.68%, and 78.48%, respectively. The accuracy (84.81%) of the dual-time-point ^18^F-FDG PET/CT model was significantly higher than that of nuclear medicine physicians.

**Conclusions:**

Based on dual-time-point ^18^F-FDG PET/CT images, the 3D nnU-net with a majority voting method, demonstrates excellent performance in predicting the malignant risk of GGOs. This methodology serves as a valuable adjunct for physicians in the risk prediction and assessment of GGOs.

## Background

Lung cancer stands as a predominant cause of cancer-related mortality worldwide, accounting for 18% of all cancer-related deaths [1]. Medical imaging plays an important role in the early diagnosis of the lung cancer. Especially, with the widespread adoption of low-dose lung computed tomography in lung cancer screening in recent years, the detection rate of subsolid nodules has significantly increased [2]. Subsolid nodules, also known as ground-glass nodules (GGOs), represent one of the most common manifestations found in lung cancer screening [3]. GGOs comprise pure ground-glass nodules (pGGO) and mixed ground-glass nodules (mGGO) with both ground-glass and solid densities [4, 5]. GGOs encompass a spectrum of lesions, including benign nodules, atypical adenomatoid hyperplasia (AAH), adenocarcinoma in situ (AIS), microinvasive adenocarcinoma (MIA) and invasive adenocarcinoma (IA) [6]. According to latest guideline published by the World Health Organization (WHO) in 2021, based on molecular genetic characteristics, pulmonary lesions were categorized into benign tumors, precursor glandular lesions, and pulmonary adenocarcinomas. Precursor glandular lesions encompass AAH and AIS, while pulmonary adenocarcinomas include MIA and IA [7]. Lung adenocarcinoma excludes AIS from the malignant category, categorizing it as AAH in the new classification [7]. This revised classification introduces challenges in diagnosing and categorizing precursor lesions and microinvasive cancers. Moreover, for GGOs suspected to be AIS, immediate surgical removal is not recommended. Instead, priority is given to follow-up observation until there is evident malignant transformation before considering surgical intervention in the 2023 NCCN lung cancer surveillance guidelines [8]. Therefore, accurate diagnosis and risk prediction of GGOs hold paramount clinical significance for decision-making according to the new WHO classification [9, 10].

Due to the heterogeneity, low density, and uneven density within GGOs tissue, the success rate of pathological biopsy is compromised [11, 12]. Qualitative diagnosis of GGOs through clinical biopsy is challenging [5]. GGOs may exhibit indolent growth, and current clinical practice involves periodic follow-up for diagnosis. Prolonged undiagnosed follow-up imposes substantial psychological burden and stress on patients. Achieving precise diagnosis of GGOs through imaging techniques is a current research focus. Positron emission tomography-computed tomography (PET/CT) with ^18^F-FDG, integrating PET and CT imaging, provides both anatomical and molecular information in a single scan, offering a crucial method for predicting malignant risk in pulmonary nodules [13, 14]. However, ^18^F-FDG PET/CT with visual analysis combined with semi-quantitative indicators still has some limitations in early diagnosis of GGOs [15].

Artificial intelligence has garnered significant attention for its crucial potential in diagnosing GGOs [16–18]. Most previous studies have been conducted based on CT images [19–21], and there is currently a lack of research on artificial intelligence methods for predicting the malignant risk of GGOs based on ^18^F-FDG PET/CT images, specifically in the context of the new WHO classification for GGOs. The 3D nnU-net, an automatic segmentation method based on convolutional neural networks, exhibits excellent generalization capabilities, adapting its architecture to different tasks and outperforming many existing methods in medical image processing in terms of accuracy, reliability, and efficiency [22–24]. Herein, we employed the 3D nnU-net with a majority voting method to predict the malignant risk of GGOs according to the new WHO classification based on dual-time-point ^18^F-FDG PET/CT images.

## Methods

### Patient selection

In this retrospective single-center study, we recruited 316 patients with GGOs who underwent dual-phase ^18^F PET/CT imaging at the department of nuclear medicine in the First Affiliated Hospital of China Medical University between June 1, 2015, and July 20, 2023. The study focused on patients exhibiting CT imaging characteristics indicative of GGOs. This research received approval from the institutional ethics committee for retrospective analysis and did not require informed consent. Inclusion criteria were as follows: (1) Stage I lung adenocarcinoma; (2) Pulmonary nodules presenting as GGOs; (3) Lesion diameter within the range of [5 mm, 30 mm]; (4) Availability of postoperative pathological diagnosis, and the time interval between PET/CT examination and pathology sample acquisition was less than 90 days, or patients with a clinical follow-up with CT exceeding 3 years. Exclusion criteria were: (1) Antitumor therapy (including neoadjuvant chemotherapy); (2) Incomplete clinical or imaging data.

### Datasets

A preliminary cohort study was conducted on 311 patients with GGOs who underwent ^18^F-FDG PET/CT scans using Siemens Biograph MCT. The scans were performed 60 min after intravenous injection of 5.5 MBq/kg ^18^F-FDG. Each bed position acquired ^18^F-FDG PET/CT data over a 90-second interval. Thin-section CT images of the lungs were obtained without contrast. A delayed scan was conducted 120 min after the initial scan. The voxel size for dual-phase PET was set at 4 mm × 4 mm × 4 mm, and CT voxel size was 0.68 mm × 0.68 mm × 0.7 mm. All scans were supervised by an experienced nuclear medicine technologist with 20 years of chest imaging expertise. The DICOM files were processed using LIFEX software (https://www.lifexsoft.org/) for delineation of GGOs in both dual-phase PET and CT images. For PET images, a semi-automatic delineation was performed using the Threshold 41% tool. For CT images, manual delineation was carried out on lung window settings (window level: -450, window width: 1500) using the pencil 2D tool for layer-by-layer delineation.

**Fig. 1:**
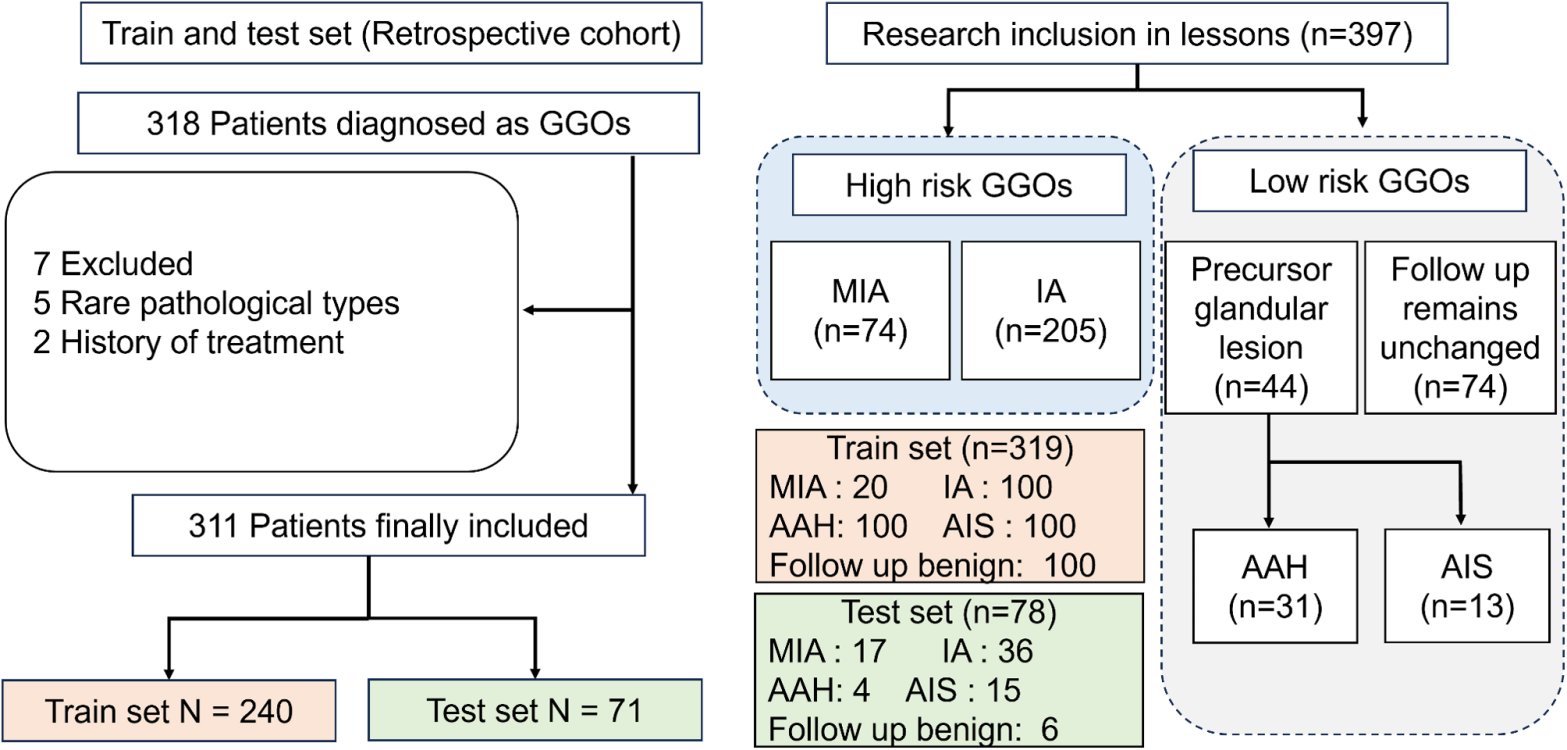
A flowchart of the patients included and excluded, a grouping diagram of different types of GGOs in this study

Based on follow-up and pathological data, the collected data were divided into two groups: the low-risk nodule group (precursor glandular lesion, follow up remains unchanged) and the high-risk nodule group (MIA, IA). Following the initial ^18^F-FDG PET/CT examination, patients underwent regular lung CT scans during a 3-year follow-up period. For pulmonary GGOs within this follow-up, if there were no changes in the nodule diameter and composition, the nodule was classified as low risk. The final study included a total of 397 GGOs, comprising 118 in the low-risk group and 279 in the high-risk group. The specific number of nodules for each category is detailed in Fig. 1.

### Method

**Fig. 2:**
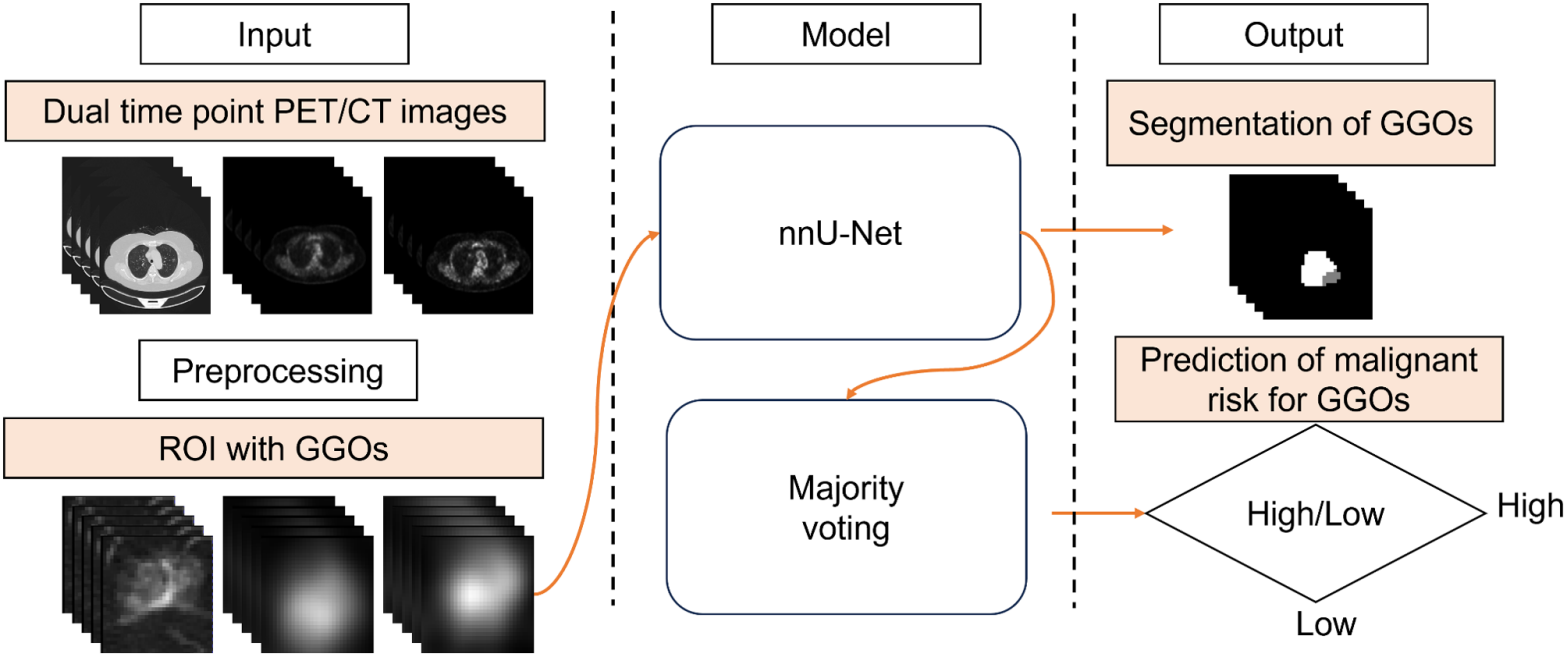
The workflow of the model for segmenting GGOs and predicting malignant risk

The workflow of the model is depicted in Fig. 2 and comprises two primary components: data preprocessing, segmentation GGOs and prediction of malignant risk for GGOs. All experiments were conducted on a single Nvidia GeForce RTX 3090. The model was constructed using PyTorch 1.4.3 and Python 3.8.

#### Data preprocessing

In the data preprocessing phase, several key steps were undertaken to ensure uniformity and relevance across ^18^F-FDG-PET, delayed-phases PET, and thin-section CT images. The ^18^F-FDG-PET, delayed-phases PET, and thin-section CT scans were resampled to achieve consistency in voxel dimensions. The resampling method employed was trilinear interpolation, a technique commonly used to estimate voxel values at non-grid positions based on surrounding grid points. This ensured alignment and uniformity across the different imaging modalities. The central points of the GGOs were identified, and three-dimensional blocks (32 × 32 × 32) were extracted from ^18^F-FDG-PET, delayed-phases PET, and CT images centered around these points. This block size was chosen to focus on the GGOs, removing irrelevant areas outside the region of interest. The CT images were subjected to windowing with a window width of 1500 and a window level of -450. This specific setting was chosen to enhance the visualization of pulmonary details within the CT images, ensuring clarity and highlighting relevant anatomical structures. The entire dataset, encompassing ^18^F-FDG-PET, delayed-phases PET, and CT images, underwent a z-score standardization process. This involved subtracting the mean voxel value from each voxel and then dividing by the standard deviation. The z-score standardization ensured that each modality had a mean of 0 and a standard deviation of 1, facilitating model convergence during subsequent training and analysis.

#### Architecture of model

**Fig. 3:**
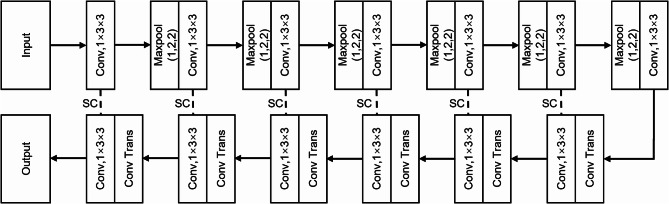
The architectural framework of the 3D nnU-Net comprises an encoder and a decoder intricately linked through skip connections

The classification network for the GGOs is depicted in Fig. 3. The input consists of the 3-channel ROI of the dual-phase ^18^F-FDG PET images and thin-section lung CT images, which were extracted in the previous step. The output of the network provides the GGOs segmentation (voxel-wise predicted labels of invasive GGOs and inert GGOs with the same imaging resolution as the input images). The primary architecture of the network is a 3D U-net [25], which belongs to the 3D nnU-net framework [26]. The 3D nnU-net is widely recognized for its high performance on 23 public datasets. It is a deep learning-based segmentation technique that has been extensively employed for medical imaging segmentation tasks and has demonstrated superiority over numerous existing approaches without requiring manual intervention. The network is composed of an encoder-decoder structure with skip connections linking the two pathways. The encoder includes 5 levels of convolutional layers with strided convolution of the same down-sampling rate. The decoder follows the same design that incorporates transpose convolution up-sampling on the concatenated up-sampled features from the lower level and the skip features from the encoder branch at the same level. After every convolution operation, Leaky ReLU with a slope of 0.01 [27] and batch normalization [28] were applied after every convolution operation. The result of the 3D nnU-net is input into the majority voting module, a straightforward yet effective classification algorithm. High-risk GGOs component prediction voxels exceeding 50% of total voxels indicate malignancy, with values below 50% classifying the lesion as low risk. The assessment of malignancy risk in the testing set (high-risk GGOs or low-risk GGOs) is further determined.

#### Training and testing details

A loss function in machine learning and deep learning estimates the disparity between predicted and actual values. It quantifies predictive accuracy during model training, guiding parameter optimization for enhanced performance. Given the use of the 3D nnU-net model for segmenting nodule volumes in this study, the loss function combines Dice loss and cross-entropy loss. Loss computation spans the entire tumor region, yielding the Dice coefficient to assess segmentation outcomes. The 3D nnU-net employs stochastic gradient descent with an initial learning rate of 0.01 and Nesterov momentum of 0.99. Training consists of 1000 epochs, with each epoch’s sample size equal to the total training set samples. Data augmentation is applied to each patch during training. The optimal model, determined post-convergence of training loss, is applied to the testing set, generating segmentation results for high-risk and low-risk GGOs components.

### Statistical analysis

For the high risk GGOs and low risk GGOs groups, significant differences were assessed by the chi-square test for categorical variables and the t-test for continuous variables. To assess the performance of the model across various modalities, three models were trained: a thin-section lung CT model, an early-phase ^18^F-FDG PET/CT model, and a dual-time-point ^18^F-FDG PET/CT model, all validated on the testing set. All models adopted the same 3D nnU-net architecture, with adjustments made to the input and layer structures based on the number of modalities in the input images. Simultaneously, to assess the clinical applicability of the models, we compared the results with those of three nuclear medicine diagnostic physicians. These physicians (resident, junior and expert), possessed over 2, 5, and 10 years of PET/CT interpretation experience, respectively. After anonymizing the imaging data, the diagnostic physicians independently interpreted the nature of pulmonary nodules in thin-section lung CT, early-phase PET, and delayed-phase PET images. They subsequently integrated the findings from all three modalities for a comprehensive qualitative assessment of pulmonary nodules. The final qualitative results were compared with the predictions of the dual-time-point ^18^F-FDG PET/CT model on the testing set. Performance metrics, including accuracy, specificity, sensitivity, positive predictive value (PPV), negative predictive value (NPV), F1 score, and the area under the ROC curve, were employed to evaluate model efficacy. All statistical analyses were performed using Python (version 3.8).

## Results

### Characteristics of patients with GGOs

This study enrolled a total of 311 patients with 397 nodules. Clinical data and nodule characteristics were subjected to analysis, encompassing variables such as gender, age, smoking history, family history of lung cancer, nodule size, nodule location, maximum standardized uptake value (SUV_max_) in the early and delayed phases. The baseline clinical features of each patient group are presented in Table 1. Among the patients, 198 were female, constituting 63.7% of the cohort; 247 were non-smokers, representing 79.4%; and 255 had no family history of lung cancer, accounting for 82%. The mean age of the patients was 60.46 years. There were 279 instances of high-risk GGOs, including 74 MIA cases and 205 IA cases, and 118 instances of low-risk GGOs, comprising 31 AAH cases, 13 AIS cases, and 74 cases with unchanged follow-up lesions. The dataset was randomly divided into a training set comprising 239 patients (318 lesions) and a testing set comprising 72 patients (79 lesions), Clinical characteristics of patients in both sets are summarized in Table 1. Statistical comparisons between the training set and testing set involved the chi-square test for categorical variables and the t-test for continuous variables. The obtained P-values for each clinical feature in both sets were all above 0.05, indicating an absence of statistically significant differences in the distribution of clinical characteristics between the two sets.

**Table 1:** Clinical characteristics for training and testing sets

		Training set (*n* = 239, m = 318)	Testing set (*n* = 72, m = 798)	*p*
Gender, n	Male	84	29	
	Female	155	43	0.56
Age (years, mean)		60.87	59.07	0.17
Smoking history, n	Yes	48	16	
	No	191	56	0.82
Lung cancer family history, n	Yes	41	15	
	No	198	57	0.59
GGOs risk Stratification	High risk	226	53	
	Low risk	92	26	0.58
GGOs Position	Left upper lobe	84	18	
	Left lower lobe	41	6	
	Right upper lobe	110	29	
	Right middle lobe	26	7	
	Right lower lobe	57	19	0.53
Early-phase PET SUV_max_ (kBq/ml/MBq/kg)		2.42	1.99	0.13
Delayed-phase PET SUV_max_ (kBq/ml/MBq/kg)		3.03	2.46	0.17
GGOs diameter		17.88	16.67	0.20

### GGOs segmentation and prediction malignant risk

**Fig. 4:**
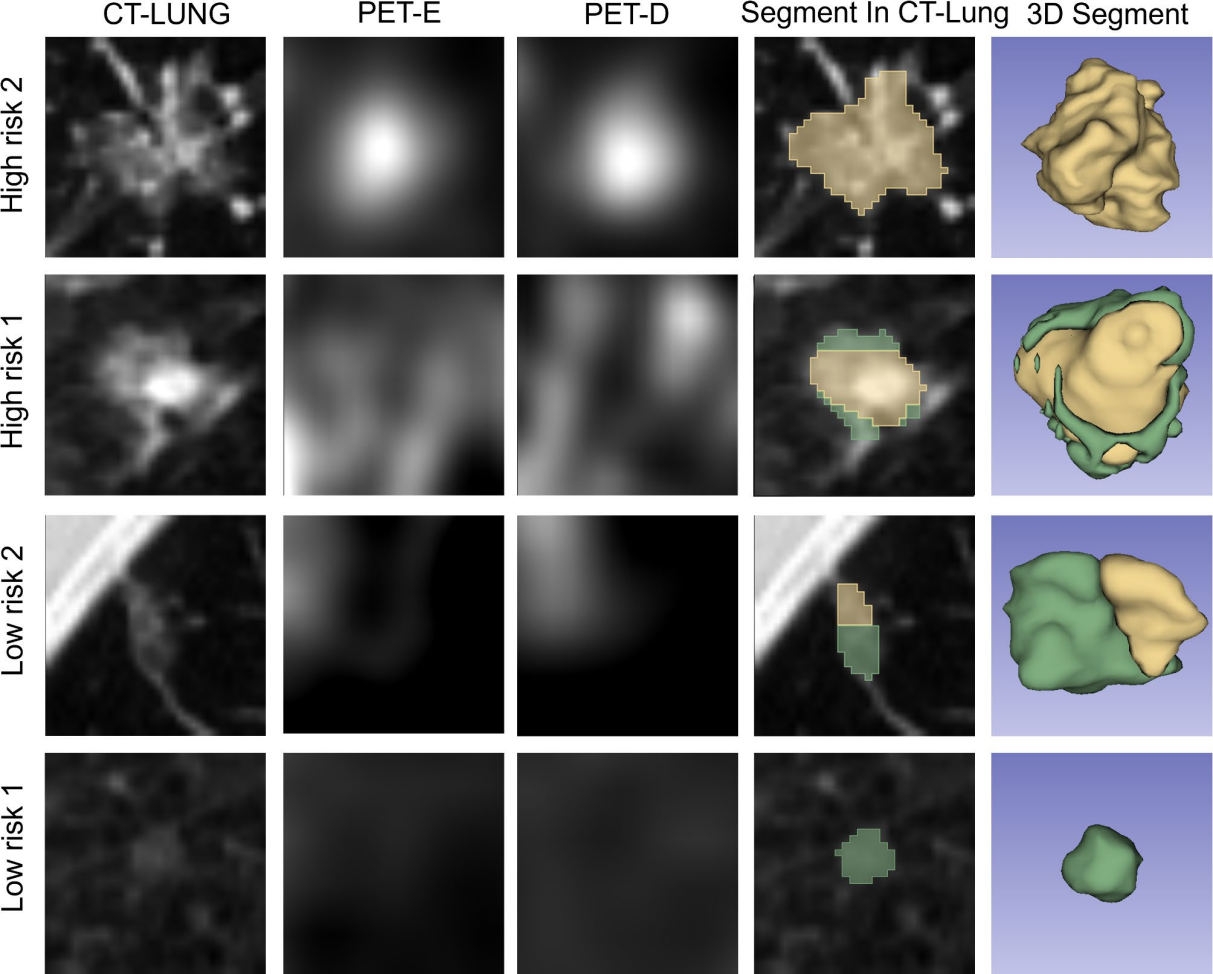
Segmentation Results of High-Risk and Low-Risk GGOs High risk1 and High risk2 represent high-risk nodules, while Low risk1 and Low risk2 depict low-risk nodules. “Segment In CT-Lung” displays the segmentation results overlaid on thin-section lung CT images, and “3D Segment” provides a three-dimensional visualization of the segmented regions. In the visualizations, the green areas represent the low-risk regions segmented by the model, while the yellow areas indicate the high-risk regions segmented by the model

The Dice score for the dual-time-point ^18^F-FDG PET/CT model was 0.84 ± 0.02, indicating a close alignment between the model’s segmentation results and the labels. The model ultimately provides segmentation results for GGOs on thin-section lung CT, divided into two components: one for the segmentation results of low-risk areas within the nodules and the other for the segmentation results of high-risk areas within the nodules. The segmentation efficacy of the dual-time-point ^18^F-FDG PET/CT model is illustrated in Fig. 4. In Low risk 1 and 2, the proportion of model-segmented high-risk areas does not exceed the cutoff value, hence predicting these nodules as low-risk. Conversely, in High risk 1 and 2, the proportion of model-segmented high-risk areas surpasses the cutoff value, leading to their prediction as high-risk nodules. After employing a majority voting method on the segmentation results output, among the testing set samples, 49 were predicted as high-risk nodules with a proportion exceeding the cutoff value, while 30 were classified as low-risk nodules falling below the cutoff value.

### Performance of the multi-modal model

**Fig. 5:**
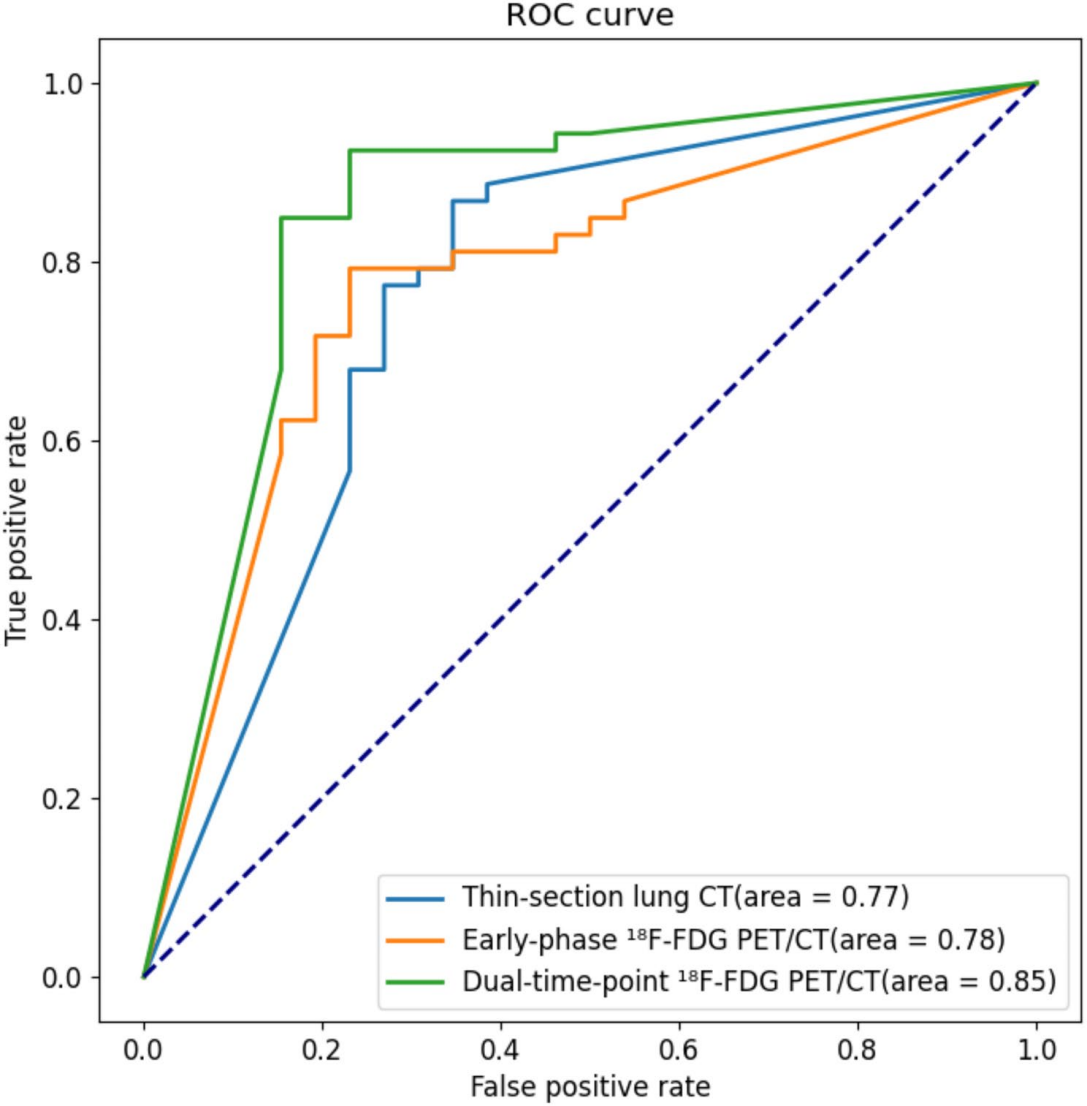
Performance of 3D nnU-net in the prediction of high risk and low risk. AUCs for the thin-section lung CT model (orange curve), the early-phase ^18^F-FDG PET/CT model (green curve) and the dual-time-point ^18^F-FDG PET/CT model (blue curve) were 0.77, 0.78, and 0.85, respectively

A combination of various imaging modalities resulted in the construction of three models: the thin-section lung CT model, early-phase ^18^F-FDG PET/CT model, and dual-time-point ^18^F-FDG PET/CT model. The diagnostic performance indicators and ROC curves for each model on the testing set are presented in Table 2; Fig. 5. Comparing these models with the thin-section lung CT model, it is evident that, with the addition of PET-E and PET-D images, the performance of each model sequentially improves. The accuracy and area under the ROC curve for the thin-section lung CT model are 73.42% and 0.77, respectively. The early-phase ^18^F-FDG PET/CT model demonstrates an accuracy of 78.48% and an area under the ROC curve of 0.78. Notably, the dual-time-point ^18^F-FDG PET/CT model achieves the highest performance metrics, with an accuracy of 84.81% and an area under the ROC curve reaching 0.85.

**Table 2:** The performance of the thin-section lung CT model, the early-phase ^18^F-FDG PET/CT model and the dual-time-point ^18^F-FDG PET/CT model was assessed using 3D nnu-net within the testing set

	Accuracy	Specificity	Sensitivity	PPV	NPV	F1 score	AUC
Thin-section lung CT	73.42%	73.08%	73.58%	84.78%	57.57%	0.79	0.77
Early-phase ^18^F-FDG PET/CT	78.48%	76.92%	79.25%	87.50%	64.52%	0.83	0.78
Dual-time-point ^18^F-FDG PET/CT	84.81%	84.62%	84.91%	91.84%	73.33%	0.88	0.85

### Comparison of assessment results with clinical doctors

Comparison of the interpretation results from three nuclear medicine diagnostic physicians with the predictive outcomes of the dual-time-point ^18^F-FDG PET/CT model for the testing set is presented in Table 3. The diagnostic accuracy of the dual-time-point ^18^F-FDG PET/CT model was 84.81%. In comparison, the accuracy for resident physician, junior physician, and expert physician was 67.09%, 74.68%, and 78.48%, respectively. The diagnostic accuracy of the dual-time-point ^18^F-FDG PET/CT model significantly surpassed that of the nuclear medicine diagnostic physicians. The diagnostic sensitivity of the dual-time-point ^18^F-FDG PET/CT model was 84.91%, whereas the sensitivity for resident physician, junior physician, and expert physician was 58.49%, 73.58%, and 75.47%, respectively. Notably, the diagnostic sensitivity of the dual-time-point ^18^F-FDG PET/CT model was markedly higher than that of the nuclear medicine diagnostic physicians.

**Table 3:** Comparison of the performance between nuclear medicine physicians’ assessments and the dual-time-point ^18^F-FDG PET/CT model

	Accuracy	Specificity	Sensitivity	PPV	NPV	F1 score	AUC
Resident physician	67.09%	84.62%	58.49%	88.57%	86.67%	0.70	0.72
Junior physician	74.68%	76.92%	73.58%	86.67%	58.82%	0.80	0.75
Expert physician	78.48%	84.62%	75.47%	90.91%	62.86%	0.82	0.80
Dual-time-point ^18^F-FDG PET/CT	84.81%	84.62%	84.91%	91.84%	73.33%	0.88	0.85

## Discussion

In this study, we employed the 3D nnU-net with a majority voting method to predict the malignant risk of GGOs according to the new WHO classification based on dual-time-point ^18^F-FDG PET/CT images. The 3D nnU-net architecture, initially proposed by Isensee et al. [26], is a deep learning segmentation method that automatically configures itself, covering preprocessing, network architecture, model training, and post-processing to address any new task. Several studies have also employed the 3D nnU-net for classification tasks. Yin et al. [22] utilized it to differentiate pelvic and sacral osteosarcoma from Ewing’s sarcoma, Cheng et al. [23] used it for predicting the molecular subtypes of posterior fossa ependymomas, and Cai et al. [24] applied it to classify extramural vascular invasion status. In each case, they achieved excellent performance. our study employs the 3D nnU-net in conjunction with a majority voting approach to predict the malignant risk of nodules. This involves the automated segmentation of GGOs on dual-time-point ^18^F-FDG PET/CT images, followed by classification via majority voting without manual intervention, forming an end-to-end process. Considering that deep learning models typically require large datasets, this voxel prediction method based on the majority principle greatly optimizes the accuracy of this classification task, enhancing model stability and efficiency [29]. With this model, the automatic discrimination of GGOs malignant risk is achieved by inputting raw images without manual delineation or any intervention. Furthermore, it can extract contextual information from consecutive slices, better capturing spatial features in lung nodules [30]. Additionally, the multi-scale feature extraction of 3D nnU-net enables it to adapt to nodules of different sizes, eliminating the disadvantages of conventional models in predicting small lung nodules [31]. Finally, 3D nnU-net reduces overfitting and training time through weight fine-tuning via transfer learning, improving algorithm efficiency and achieving high model accuracy with a smaller sample size.

In terms of GGOs segmentation, the obtained Dice coefficient of 0.84 surpasses previously reported DICE coefficients for GGOs segmentation using CT (Dice scores ranging from 0.73 to 0.82) [32, 33], underscoring the clinical potential of the model in GGOs segmentation. The interpretation of the segmentation results reveals that the model delineates the 3D morphology of lung nodules, indicating the location and proportion of high-risk and low-risk regions. The high-risk areas are concentrated in the solid density, lobulation, spiculation, and pleural traction areas of lung nodules, showing increased FDG uptake and delayed-phases PET imaging uptake (as shown in Fig. 4). This matches the malignant characteristics judged in clinical practice [34, 35], providing support for the improved efficacy of the model.

Regarding model predictive performance, the dual-time-point ^18^F-FDG PET/CT model in this study achieved an accuracy, sensitivity, specificity, and AUC of 84.81%, 84.91%, 84.62%, and 0.85, respectively. Compared to the early-phase ^18^F-FDG PET/CT model and thin-section lung CT model, it outperformed in all metrics, and with the addition of delayed-phases PET, the model performance sequentially increased. Particularly, the inclusion of delayed-phases PET demonstrated the greatest enhancement in model performance, indicating its value in predicting GGOs malignant risk. Additionally, when comparing our dual-timepoint PET/CT model with existing CT-based methods for GGO malignant risk assessment (AAH and AIS vs. MIA and IA) reported by Xu et al. [36] (accuracy: 82.2%, AUC: 0.831) and Wang et al. [37] (accuracy: 73.4%, AUC: 0.813), our approach demonstrated increased accuracy and AUC. This gain may be related to the upregulation of glucose consumption by malignant cells to obtain more proliferative energy, leading to the concentration of ^18^F-FDG in delayed-phases PET [38, 39]. In contrast to other convolutional neural networks, the diagnostic performance of the dual-time-point ^18^F-FDG PET/CT model is more balanced, with other convolutional neural networks exhibiting lower overall specificity, indicating limited efficacy in predicting low-risk GGOs. Furthermore, when compared with nuclear medicine diagnostic physicians, the dual-time-point ^18^F-FDG PET/CT model achieved higher diagnostic efficacy, especially surpassing more experienced physicians, confirming its clinical application value. Previous studies using radiomics and deep learning methods for GGOs classification under early-phase ^18^F-FDG PET/CT achieved an accuracy of around 80% [40, 41]. However, these models showed significant variations in sensitivity and specificity, and their results were not validated against the diagnostic efficacy of clinical physicians [42, 43].

Clinically, the interpretation of PET/CT images for pulmonary GGOs relies on subjective judgment supported by the experience of diagnostic physicians. Comparing the model with manual readings, significant differences in diagnostic efficacy were observed between low-experience physicians, high-experience physicians, and the model. Moreover, the majority of GGOs exhibits characteristics of indolent growth, with approximately 20% of pure GGOs and 40% of mixed GGOs gradually increasing or adding solid components over time, while the majority remains unchanged for several years. For distinguishing between growing and non-growing GGOs, a reasonable benchmark is a volume doubling time (VDT) of approximately 600 to 900 days for pure GGOs and 300 to 450 days for mixed GGOs during a 3-year follow-up observation [44]. The specific time intervals and frequency of follow-up in clinical practice vary among individuals, requiring physicians to perform comparative analyses of nodules at different time points to determine their nature.

In lung cancer screening, the occurrence of multifocal GGOs is highly prevalent [45]. For cases involving multiple GGOs that necessitate clinical intervention, guidelines recommend a preference for surgical procedures. The principle is to prioritize the treatment of primary lesions while addressing secondary lesions [46]. But performing pathological evaluation on every lesion is neither feasible nor resource-efficient. Furthermore, the presence of multiple lesions within a single patient may introduce a “clustering effect,” potentially leading to overfitting to patient-specific characteristics in predictive models [47]. Clarifying these issues through future research could refine risk stratification and guide more nuanced clinical decision-making for multifocal GGO management.

However, it is essential to acknowledge certain limitations in this study. The use of a retrospective single-center dataset resulted in a relatively small sample size. Further extensive prospective multicenter research is necessary to evaluate the generalizability of the established model in clinical practice.

## Conclusions

In conclusion, our study revealed the effectiveness of utilizing dual-time-point ^18^F-FDG PET/CT images along with the 3D nnU-net and a majority voting method for predicting the malignant risk of GGOs based on the new WHO classification. This methodology serves as a valuable adjunct for physicians in the risk prediction and assessment of GGOs.

## Data Availability

No datasets were generated or analysed during the current study.

## Abbreviations

GGOs: Ground-glass nodules
AAH: Atypical adenomatoid hyperplasia
AIS: Adenocarcinoma in situ
MIA: Microinvasive adenocarcinoma
IA: Invasive adenocarcinoma
WHO: World Health Organization
PET/CT: Positron emission tomography-computed tomography
PPV: Positive predictive value
NPV: Negative predictive value
SUV_max_: Maximum standardized uptake value
